# Transcriptomic Analysis of *Campylobacter jejuni* Following Exposure to Gaseous Chlorine Dioxide Reveals an Oxidative Stress Response

**DOI:** 10.3390/ijms26073254

**Published:** 2025-04-01

**Authors:** Gretchen E. Dykes, Yiping He, Tony Jin, Xuetong Fan, Joe Lee, Sue Reed, Joseph Capobianco

**Affiliations:** Characterization and Interventions for Foodborne Pathogens Research Unit, Eastern Regional Research Center, Agricultural Research Service, United States Department of Agriculture, USDA-ARS-ERRC, 600 East Mermaid Lane, Wyndmoor, PA 19038, USAtony.jin@usda.gov (T.J.); xuetong.fan@usda.gov (X.F.); joe.lee@usda.gov (J.L.); suebug2727@gmail.com (S.R.); joseph.capobianco@usda.gov (J.C.)

**Keywords:** *Campylobacter jejuni*, RNA-Seq, genome sequencing, gaseous chlorine dioxide, oxidative stress response, antimicrobial activity, gene expression, transcription

## Abstract

Gaseous chlorine dioxide (ClO_2_) is a potent antimicrobial agent used to control microbial contamination in food and water. This study evaluates the bactericidal activity of gaseous ClO_2_ released from a sodium chlorite (NaClO_2_) pad against *Campylobacter jejuni*. Exposure to a low concentration (0.4 mg/L) of dissolved ClO_2_ for 2 h resulted in a >93% reduction of *C. jejuni*, highlighting the bacterium’s extreme sensitivity to gaseous ClO_2_. To elucidate the molecular mechanism of ClO_2_-induced bactericidal action, transcriptomic analysis was conducted using RNA sequencing (RNA-seq). The results indicate that *C. jejuni* responds to ClO_2_-induced oxidative stress by upregulating genes involved in reactive oxygen species (ROS) detoxification (*sodB*, *ahpC*, *katA*, *msrP*, and *trxB*), iron transport (*ceuBCD*, *cfbpABC*, and *chuBCD*), phosphate transport (*pstSCAB*), and DNA repair (*rdgB* and *mutY*). Reverse transcription-quantitative PCR (RT-qPCR) validated the increased expression of oxidative stress response genes but not general stress response genes (*spoT*, *dnaK*, and *groES*). These findings provide insights into the antimicrobial mechanism of ClO_2_, demonstrating that oxidative damage to essential cellular components results in bacterial cell death.

## 1. Introduction

*Campylobacter* is the causal agent of Campylobacteriosis, a diarrheal disease, typically contracted by consuming raw or undercooked poultry [1]. In the year 2024 in the United States, there were five outbreaks of *Campylobacter* associated with 27 *C. jejuni* isolates, of which 67% had clinically important antimicrobial resistance [2]. Due to the high occurrence of antimicrobial resistance in *C. jejuni* clinical isolates and retail meat isolates [3,4], it is critical to investigate new antimicrobial interventions for *C. jejuni* contamination on meat.

Gaseous chlorine dioxide (ClO_2_) treatment has previously been established by the Food and Drug Administration as an effective disinfection agent for poultry, fruits, and vegetables (21 CFR §173.300). Chlorine dioxide has strong antimicrobial activity against a variety of pathogens; for example, treatment with 5 mg/L gaseous ClO_2_ (60 min) reduced populations of *Pseudomonas aeruginosa* (Gram-negative) by 5 log, *Staphylococcus aureus* (Gram-positive) by 6 log [5], treatment with gaseous ClO_2_ (<28 mg/L, 2.5 h) reduced *Salmonella enterica* serotype Typhimurium by almost 2 log [6], and treatment with 10 mg/L aqueous ClO_2_ (15 min) reduced *Escherichia coli* (*E. coli*, Gram-negative) by 5.5 log [7]. In poultry processing, a study found that treatment with 50 mg/L of ClO_2_ reduced the numbers of *Campylobacter* and *E. coli* on carcasses compared to untreated controls [8]. ClO_2_ has been noted as a strong oxidant and therefore may cause cell death through oxidation of nucleic acids and amino acids [9]. Previous work has demonstrated that treatment with gaseous ClO_2_ increased cellular reactive oxygen species (ROS) in *E. coli*; however, in that study, oxidative stress response genes were not upregulated in treated samples [10].

*C. jejuni* (Gram-negative) hosts a variety of mechanisms to cope with oxidative stress. In the classical oxidative stress response, superoxide dismutase (SodB) converts reactive oxygen species (ROS) into oxygen and hydrogen peroxide (2O_2_^−^+2H^+^→H_2_O_2_+O_2_), and then hydrogen peroxide is cleared from cells by a catalase (KatA) (2H_2_O_2_→2H_2_O+O_2_), alkyl hydroperoxide reductase (Ahp) (ROOH+NADH+H^+^→ROH+NAD^+^+H_2_O), and/or peroxiredoxin (Tpx, Bcp). In addition, to repair damage from oxidation, methionine disulfide reductases (MsrP) reduce oxidized methionine groups by adding an electron donated from thioredoxin (TrxA), regenerated by thioredoxin-disulfide reductase (TrxB) [11]. These oxidative stress responses are regulated by the PerR (peroxide stress regulator) system, which is a homolog of the ferric uptake regulator (Fur) system. The Fur repressor regulates iron uptake genes/systems, including CeuB, ExbBDE, CfrAB, CtuA, and ChuA, etc. Iron transport systems play important roles in acquiring the necessary iron. Iron is used for bacterial growth but also functions as a cofactor in oxidative stress defense systems [12]. PerR and Fur overlap as regulators of the PerR system, which is unsurprising as hydroxyl radicals are generated when Fe^3+^ is reduced to Fe^2+^ in the Fenton reaction, necessitating protection from oxidative damage. Fur represses transcription using an Fe^2+^ cofactor [13]; however, modulation of the PerR repressor is unclear. As a microaerophile, *C. jejuni* is highly sensitive to oxidative stress and to other general stresses, such as nutrient deprivation, environmental changes (e.g., temperature, PH, humidity, osmotic pressure, and atmospheric gas composition), and DNA damage. Therefore, the oxidative stress response of *C. jejuni* is robust and facilitates investigation of oxidative stressors.

While gaseous ClO_2_ has been demonstrated as an effective pathogen intervention in foods, it is still poorly understood how gaseous ClO_2_ inactivates bacterial cells. Previous work showed that treatment with ClO_2_ increased ROS; however, in that study, oxidative stress response genes were not upregulated in treated samples, as it measured a limited number of genes by qRT-PCR [10]. Accordingly, this study aims to elucidate the mechanism by which ClO_2_ inactivates cells. We performed genome-wide RNA sequencing (RNA-seq) and a comprehensive transcriptome analysis to reveal up- and down-regulated RNA transcripts across the entire *C. jejuni* genome following exposure to gaseous ClO_2_.

## 2. Results and Discussion

### 2.1. Dissolved ClO_2_ in Water

**Figure 1:**
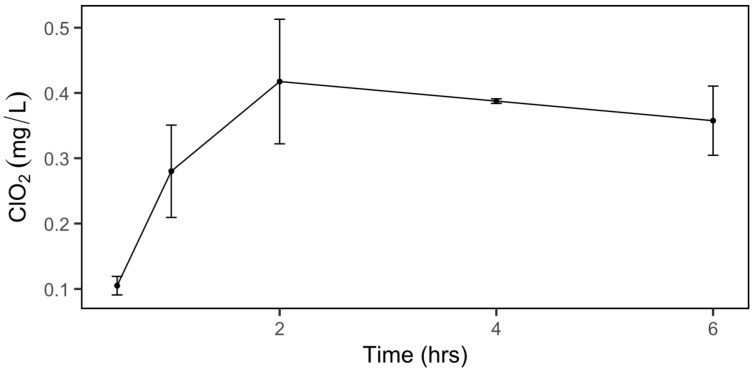
Concentrations of dissolved ClO_2_ in water. The values of ClO_2_ concentration are the means of two replicates and two measurements per sample. Error bars indicate standard deviations of the means.

To determine the level of ClO_2_ gas released from an NaClO_2_ pad, a time course experiment that measured absorbed ClO_2_ in the water was performed with duplicate samples incubated in an air-tight container with an NaClO_2_ pad for 0, 0.5, 1, 2, 4, or 6 h. The concentration of dissolved ClO_2_ gas in water increased until the 2 h mark, after which it remained steady at about 0.4 mg/L ClO_2_ (Figure 1). We used water instead of brucella broth as a solution for the measurement, because ClO_2_ reacted with the components in the brucella broth and resulted in inaccurate readings.

### 2.2. Exposure of C. jejuni to Gaseous ClO_2_

Next, the length of exposure to ClO_2_ needed to stress and kill *C. jejuni* cells was determined by exposing a late-log phase culture (9.2 × 10^7^ CFU/mL) to gaseous ClO_2_ in an air-tight container for up to 4 h. When *C. jejuni* cells were exposed to gaseous ClO_2_, the number of viable cells decreased after 1 h of exposure, and 93% (sample size n = 12, *p*-value < 0.001) of the cells were killed at 2 h. Therefore, we chose to use a 1 h exposure period in our qRT-PCR and RNA-Seq experiments to capture a timepoint when the cells would be stressed, but not dead.

### 2.3. RNA-Seq

To understand how *C. jejuni* responds to ClO_2_ and to elucidate the mechanism by which ClO_2_ causes cell death, we sequenced RNA transcripts of triplicate *C. jejuni* cultures exposed to gaseous ClO_2_ for one hour and compared them to transcripts of untreated samples. All samples had between 22 to 24.5 million read pairs, 94% of base pairs had a Q score greater than 30, and 68–73% of reads aligned to the reference genome. The genes and their annotated proteins, differentially expressed in gaseous ClO_2_ -treated vs. untreated cells, are shown in Supplementary Table S1.

- Oxidative stress response

**Figure 2:**
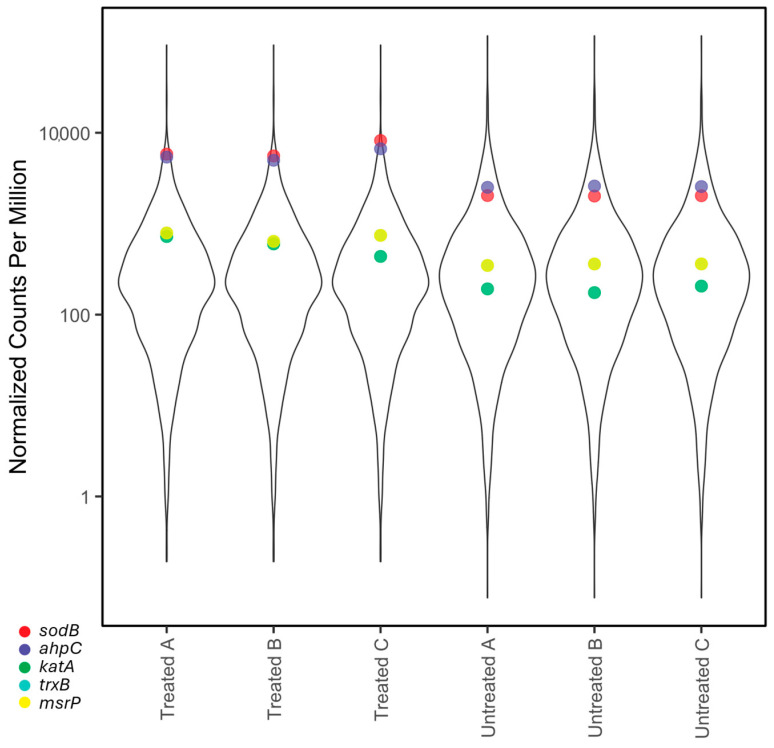
Violin plot showing the distribution of normalized counts per million for each sample and expression of selected genes. Here, the *y*-axis shows the normalized counts per million of each transcript (log-transformed), and the *x*-axis shows the distribution of transcripts for each sample, where wider plots had more transcripts at that level of transcription. The points indicate the normalized counts per million for the specific gene (color coded).

**Figure 3:**
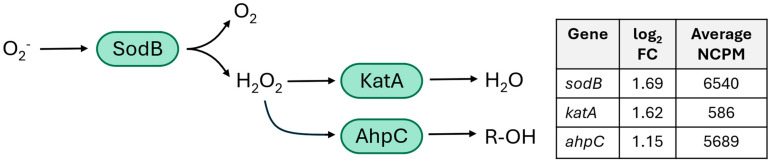
Genes upregulated in *C. jejuni* cells treated with gaseous ClO_2_ related to the typical oxidative stress response. The role of proteins in the oxidative stress response are depicted in the flow chart in green. The log2 fold change (FC) in expression in treated cells compared to untreated cells is listed in the table for each gene related to the oxidative stress response. All genes were significantly upregulated, with a Benjamini–Hochberg-adjusted (BH adj.) *p*-value < 0.001.

Genes involved in an oxidative stress response had significantly higher expression in treated samples compared to untreated samples and were among the most highly expressed transcripts (Figure 2). *sodB* was the most highly expressed transcript with differential expression in treated samples and had significantly higher expression in treated samples than in untreated samples (Figure 2 and Figure 3). *sodB* encodes an iron superoxide dismutase, which catalyzes the conversion of superoxide into oxygen and hydrogen peroxide [11] and was previously upregulated in *C. jejuni* cells under presumed oxidative stress [14]. *katA* (a catalase) was highly expressed in treated samples (Figure 2 and Figure 3) and catalyzes the conversion of peroxide to water [15]. *katA* expression is induced by the presence of H_2_O_2_ and O_2_^−^ [11]. *ahpC* was the third most highly expressed transcript in treated samples and also had significantly higher expression in treated samples than in untreated samples (Figure 2 and Figure 3). *ahpC* encodes alkyl hydroperoxide reductase C, an antioxidant responsible for clearing peroxides from the cell [16].

b. Methionine sulfoxide reductase

**Figure 4:**
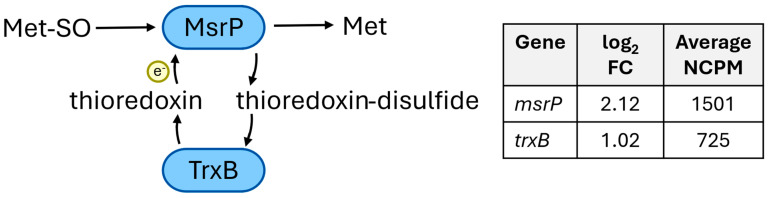
Genes upregulated in *C. jejuni* treated with gaseous ClO_2_ related to the methionine sulfoxide reductase response. The role of proteins in the methionine sulfoxide reductase stress response are depicted in the flow chart. The log2 fold change (FC) in expression in treated cells compared to untreated cells is listed in the table for each gene related to oxidative stress response. All genes were significantly upregulated, with a BH adj. *p*-value < 0.001.

*msrP*, encoding methionine sulfoxide reductase, had one of the highest-fold changes in expression in treated cells compared to untreated cells (log_2_FC = 2.12, BH adj *p* = 3.8 × 10^−6^) (Figure 4). It has been reported that MsrP repairs oxidative stress to periplasmic proteins containing methionine sulfoxide residues in *Salmonella enterica* serotype Heidelberg SL476 ([17,18], confirmed for *Campylobacter*) using thioredoxin as an electron donor [11]. In addition, thioredoxin disulfide reductase (*trxB*; log_2_FC = 1.02, BH adj *p* = 0.0007) and a TlpA disulfide reductase family protein (locus tag Q7258_08150; log_2_FC =1.23, BH adj *p* = 0.0006) were upregulated in treated samples. TrxB catalyzes the reduction of thioredoxin disulfide to thioredoxin, thus priming the cell for additional oxidative stress repair (Figure 4). The TlpA disulfide reductase family protein is a thioredoxin-like protein that was previously shown to play a role in oxidative stress response in *Neisseria gonorrhoeae* [19].

c. Iron transport response

**Figure 5:**
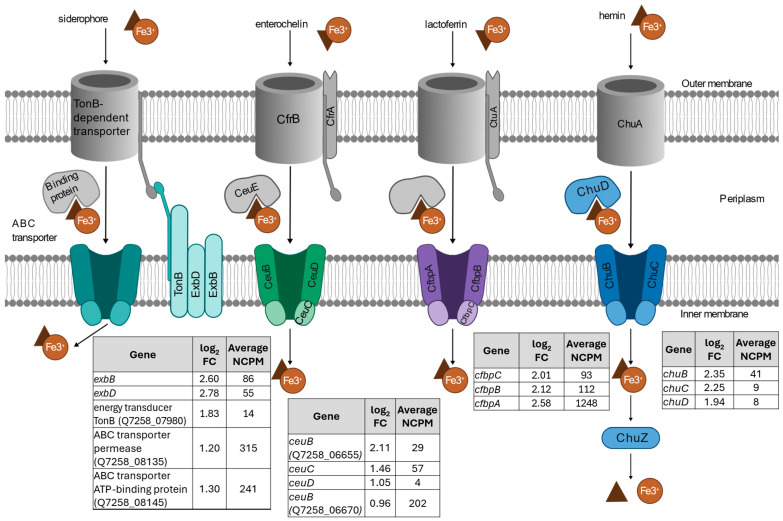
Genes upregulated in *C. jejuni* treated with gaseous ClO_2_ related to the iron transport system. The role of proteins in iron transport are depicted in the flow chart. The log2 fold change (FC) in expression in treated cells compared to untreated cells is listed in the table for each gene related to the oxidative stress response along with the average normalized counts per million (NCPM). All genes encoding proteins colored in blue, purple, green, or teal were significantly upregulated in treated cells with a BH adj. *p*-value < 0.001.

Iron siderophore transport genes were upregulated in treated samples compared to untreated samples (Figure 5). Genes related to the TonB system, including *exbD*, *exbB*, and a TonB energy transducer (locus tag Q7258_07980), were among the most highly upregulated genes in treated samples compared to untreated samples. The TonB system primarily transports iron–siderophore complexes across the bacterial outer membrane and has high affinity for siderophores [13]. In *E. coli*, TonB is regulated by the Fur repressor, which represses transcription using an Fe(II) cofactor [13]. As a strong oxidizing agent, ClO_2_ effectively oxidizes iron by converting soluble ferrous iron Fe(II) into insoluble ferric iron Fe(III), thereby lifting transcription repression by Fur and increasing the expression of TonB. In addition, *ceuB*, *ceuC*, and *ceuD* were upregulated in treated samples, though they had low expression overall compared to genes involved in superoxide decomposition (Figure 5). The Ceu system is an inner-membrane ABC transporter for Fe(III)-enterochelin uptake [20]. In *E. coli*, enterochelin (or enterobactin) was associated with the ability to reduce oxidative stress, likely because hydrolyzed enterobactin contains free hydroxyl groups with the ability to scavenge free radicals [21,22]. It is possible that enterobactin plays a similar role in the oxidative stress response in *C. jejuni.* In addition, both promoters of *ceuBCD* and *exbBD* operons in *C. jejuni* contain a hexamer Fur-binding consensus sequence [12], supporting that the upregulation of genes involved in iron acquisition was through the release of Fur repression when cells encounter Fe(II)-limiting conditions, allowing cells to efficiently acquire iron for survival.

Other iron-related and ambiguous ABC transporters were also upregulated in treated samples, including the Cfbp system (*cfbpABC*) and the Chu system (*chuBCD*) (Figure 5). The Cfbp system is an iron ABC transport system. Previous work demonstrated that CfbpA was more abundant under aerobic growth conditions in *C. jejuni*, and in that work, the authors proposed that CfbpA may aid in the oxidative stress response by ensuring sufficient iron is available for other oxidative stress response proteins which require iron co-factors [23]. In potential support of this, genes involved in hemin transport had higher expression in treated cells. For example, a Cj1386 family hemin-binding protein (locus tag Q7258_06835) was upregulated in treated cells. *chuBCD* were also significantly upregulated in treated samples. ChuA of the Chu system is likely involved in heme transport; however, *chuA* did not have significantly different expression in treated vs. untreated cells, and ChuBCD are not necessary for heme transport (and may either be redundant or not involved in heme transport [12]).

Together, these results demonstrate that cells treated with gaseous ClO_2_ upregulate genes (highlighted in color in Figure 5) related to iron transport across the cytoplasmic membrane, with specific emphasis on siderophore transporters. More iron transport genes were upregulated than any other functional category with 16 iron transport genes upregulated in treated cells. Upregulation of iron and especially siderophore transporters may benefit the cell under oxidative stress by ensuring sufficient iron is present for incorporation as a co-factor in oxidative stress response proteins [23] and by providing an enterobactin-mediated stress response [21,24].

d. Phosphate response

Genes related to a phosphate ABC transporter including *pstSCAB* had significantly higher expression in treated samples than in untreated samples. PstSCAB is a phosphate transport system and may be related to the oxidative and/or generic stress response. Transport of phosphate by the PstSCAB system is heavily regulated by the inorganic phosphate concentration and mediated by binding of phosphorylated PhoP to the promoter of the PstSCAB operon [25]. In *E. coli*, a *pst* mutant was more susceptible to reactive oxygen species than wildtype strains [26], and expression of *pstSCAB* was also upregulated after cells were exposed to chlorine [27]. In several different bacteria, activation of the Pho operon (which contains *pstSCAB* in *C. jejuni* [28]) results in increased expression of genes involved in oxidative stress response, including catalases, DNA protectants, and superoxide dismutases [29]. To defend against oxidative stress, cells utilize phosphate for repairing damaged molecules and maintaining cellular homeostasis, leading to increased uptake of phosphate from the environment and likely increasing the expression of the PstSCAB system [30].

e. DNA repair/protection

It has been established that oxidative stress damages DNA by producing reactive oxygen species (ROS) that react with DNA bases, causing strand breaks and base modifications. We found that multiple genes related to DNA repair and/or protection were upregulated in ClO_2_-treated samples. *rdgB* was upregulated in treated samples compared to untreated samples (log_2_FC = 1.03, BH adj *p* = 0.002). *rdgB* encodes a nucleoside triphosphate pyrophosphatase. In *E. coli*, RdgB hydrolyses xanthosine triphosphate (XTP) and deoxyinosine triphosphate (dITP), which are mutagenic products of purine nucleotide deamination under oxidative stress [31]. In addition, *mutY* had 1.3-fold higher expression (BH adj *p* < 0.05) in treated cells. *mutY* encodes a DNA glycosylase that helps repair oxidative DNA damage and protects cells from genetic instability [32]. Upregulated expression of *rdgB* and *mutY* in *C. jejuni* in response to gaseous ClO_2_ is likely a self-defense mechanism through increased repair of mutated/damaged DNA.

*groL* (chaperone), *groES* (co-chaperonin), and *dnaK* (chaperone) were downregulated in treated samples compared to untreated samples (log2 fold change −1.3, −1.4, and −0.7 respectively). *groES* and *groL* are on a different operon than the *fur*/*perR* regulons. A study shows that this operon was upregulated in response to increased temperature, so this operon may not be as sensitive to changes in oxidative stress. *dnaK* is found adjacent to other heat-shock proteins and may be regulated in response to heat stress.

f. qRT-PCR

**Figure 6:**
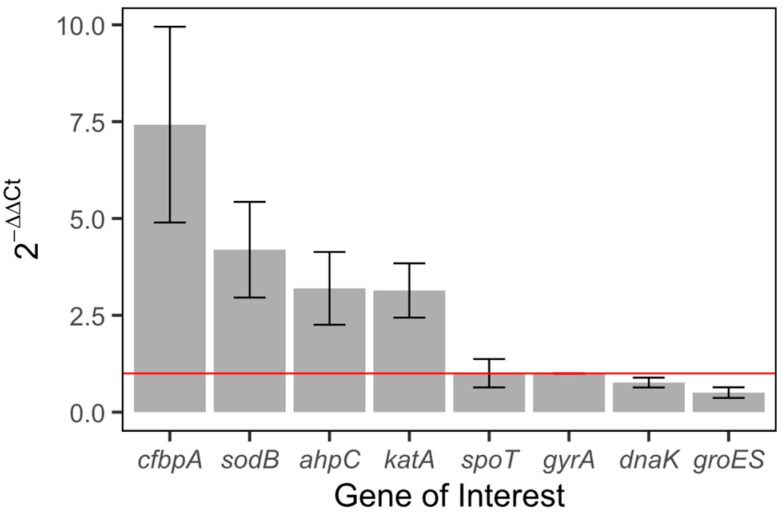
Fold change comparison of oxidative and general stress response genes compared to housekeeping gene gyrA between treated and untreated samples. Each gray bar represents the relative fold change in expression of the gene where ${relative\;fold\;change=2}^{-({\left({Ct}_{GOI}-{Ct}_{HKG}\right)}_{treated}-{\left({Ct}_{GOI}-{Ct}_{HKG}\right)}_{untreated})}$. Error bars represent the standard deviation across three replicates. An independent two group t-test resulted in a *p*-value < 0.055. The horizontal red line indicates a log2FC (fold change) of 1, or no change in gene expression.

To confirm the results of RNA-Seq, we assessed the expression of genes previously indicated in the general stress response and oxidative stress response in *C. jejuni* with qRT-PCR [14,33]. Compared to the housekeeping gene *gyrA*, *cfbpA, sodB, ahpC*, and *katA* had significantly higher expression in treated samples than in untreated samples, which parallels our findings from the RNA-Seq analysis (Figure 6). One gene, *groES,* had a lower expression than *gyrA* in treated samples than in untreated samples (independent two-group *t*-test, *p* < 0.02). *groES* was also downregulated in our RNA-Seq results. Finally, *spoT* and *dnaK* did not have significantly different expression in the qRT-PCR analysis or the RNA-Seq analysis. These results validated our RNA-Seq results and supported that cells exposed to ClO_2_ underwent an oxidative stress response.

## 3. Materials and Methods

### 3.1. Culturing Campylobacter jejuni

*C. jejuni* YH009 (S27Cj) was previously isolated from chicken thighs [34]. The strain was streaked onto a brucella plate from a frozen stock culture then incubated overnight. The next day, a loopful of colonies was inoculated into 2 mL brucella broth and then incubated overnight. Finally, 30 mL of brucella broth was inoculated with 100 µL of the overnight culture and then incubated for 16 h. All cultures were incubated at 42 °C with an EZ Campy Container System Sachet (Becton, Dickinson and Company, Franklin Lakes, NJ, USA) in an air-tight container.

### 3.2. Preparation of Chemical Pads

Five grams of sodium chlorite (NaClO_2_) powder (80% purity, Sigma-Aldrich Chemical Co., Louis, MO, USA) were dissolved in 10 mL distilled water. One ml of the solution was added to a paper pad (ca. 1.5 × 1.5 inch square). The pads were vacuum-dried at 45 °C for 12 h.

### 3.3. Measurement of Dissolved Gaseous Chlorine Dioxide

To measure chlorine dioxide released from NaClO_2_ pads, tubes containing 3 mL deionized water were placed into the same air-tight container used for the *C. jejuni* inactivation experiment containing an NaClO_2_ pad. Dissolved chlorine dioxide gas (ClO_2_) in water was measured in duplicate after exposure to an NaClO_2_ pad for 0, 0.5, 1, 2, 4, or 6 h. For each measurement, separate containers were used, and three tubes were removed from each container and directly subjected to the ClO_2_ analysis. A packet of Cl-free DPD powder (Hach Company, Loveland, CO, USA) was added to one of the 50 mL falcon tubes containing 3 mL treated water, and then two additional 3 mL aliquots were combined immediately (9 mL total) into the tube with the DPD powder. After swirling gently to mix, the total dissolved ClO_2_ was measured with a Hach colorimeter (model DR/890, Hach, Loveland, CO, USA).

### 3.4. Inactivation of C. jejuni by Gaseous ClO_2_

Overnight cultures of *C. jejuni* were gently homogenized, aliquoted into 50 mL falcon tubes (3 mL cultures/tube), and then treated with an NaClO_2_ pad adhered to the top of an air-tight container in triplicate for each timepoint. In parallel, a negative control set of samples was prepared with the overnight culture and incubated in an air-tight container with no NaClO_2_ pad attached. All samples were incubated with an EZ Campy Container System Sachet (BD) at 42 °C with no caps for 4 h with shaking at 100 rpm.

At each sampling timepoint, a 200 µL aliquot of treated and untreated *C. jejuni* culture was sampled for cell enumeration using the 6 × 6 drop plate method as described before [35]. In brief, a 1:10 serial dilution ranging from undiluted to 1 × 10^−6^ was prepared with brucella broth in a 96-well plate. Using a multi-channel pipette, 7 µL of the dilutions 1 × 10^−1^ through 1 × 10^−6^ was pipetted onto a brucella agar plate six times, resulting in six replicates of the six dilutions or 36 droplets per agar plate. Technical repeat was performed for each sample. Droplets were air-dried for approximately 15 min and then incubated with an EZ Campy Container System Sachet (BD) in an air-tight container at 42 °C overnight for cell counting.

### 3.5. Sample Treatment with Gaseous ClO_2_

A total of 90 mL of the overnight *C. jejuni* culture in the late log phase of growth were treated with gaseous ClO_2_ in three replicates. To increase the sample surface area exposed to ClO_2_ gas, 30 of 3 mL cultures were prepared in 50 mL Falcon tubes (10 cultures for each replicate) and incubated with an NaClO_2_ pad attached into the top of an air-tight container (10 cultures per container). For untreated samples, 30 mL of the culture was incubated in triplicate in a single air-tight container with no NaClO_2_ pad. All samples were incubated with an EZ Campy Container System Sachet at 42 °C with no caps for 1 h with shaking (100 rpm) to ensure maximum exposure of the cells to the gaseous ClO_2_ released from the NaClO_2_ pad.

### 3.6. Sampling and RNA Preparation

For all samples, 200 µL of culture was aliquoted for cell enumeration by the 6 × 6 drop plate method. The remaining cultures were centrifuged for 5 min at 8000× *g* at 4 °C and the pellet was immediately resuspended in 2 mL TRI-reagent and then frozen at −20 °C.

RNA was extracted from the *C. jejuni* cultures treated or not treated with gaseous ClO_2_ using the Zymo Direct-zol RNA mini-prep kit (Zymo Research, Irvine, CA, USA) according to the manufacturer’s instructions, including the DNase I treatment. During the final step of the protocol, RNA was eluted with 25 µL DNase-/RNase-free water, centrifuged (10,000× *g* for 30 s), and then was eluted a second time with 25 µL DNase-/RNase-free water, keeping the first and second eluates separate.

RNA was quantified using the Qubit BR RNA kit, according to the manufacturer’s instructions (Thermofisher, Waltham, MA, USA). To remove the remaining DNA, 1 µg RNA was diluted to 8 µL and then treated with 1 µL Amplification Grade DNase I (Invitrogen, Waltham, MA, USA), according to the manufacturer’s instructions. Finally, RNAs were diluted to 10 ng/µL with DNase-/RNase-free water.

### 3.7. RT-qPCR

Expression of genes associated with oxidative stress and general stress in *Campylobacter* [14,33] was assessed by RT-qPCR. Reverse transcription and qPCR were performed in one thermocycler run using SuperScript™ III Platinum™ SYBR™ Green One-Step qRT-PCR Kit (Invitrogen, Waltham, MA, USA). Each 20 µL reaction contained 1X Super script III RT/Platinum Taq Mix, 1X SYBR Green Reaction Mix, 5 µM forward primer, 5 µM reverse primer, 1 ng RNA, and DNase-/RNase-free water. The primer sequences are listed in Table 1. Samples were tested in duplicate for each target gene. The thermocycling program was as follows: 50 °C for 3 min, 95 °C for 5 min; followed by 40 cycles of 95 °C for 15 s, 60 °C for 1 min; and then 40 °C for 1 min. Fluorescence was read each cycle after the 60 °C annealing/elongation phase, and a standard melting curve analysis was performed following amplification. qRT-PCR results were analyzed using the ΔΔCt method with the housekeeping gene *gyrA*, in which $∆C_{t}=C_{t}^{target\;gene}-C_{t}^{housekeeping\;gene}$ and $∆∆C_{t}={∆C}_{t}^{treated\;sample}-{∆C}_{t}^{untreated\;sample}$.

### 3.8. RNA Sequencing

Purified RNA samples were submitted to SeqCenter (Pittsburgh, PA, USA) for RNA sequencing. Samples were treated with DNase I (RNase-free, Invitrogen). The Stranded Total RNA Prep Ligation with Ribo-Zero Plus kit (Illumina, Inc., San Diego, CA, USA) was used for rRNA depletion, cDNA synthesis, and library preparation. The Ribo-Zero Plus reagents bound to and enzymatically depleted the abundant rRNA (5S, 16S, and 23S rRNAs) from the total RNA. Then, the remaining mRNA was converted into cDNA. Sequencing of the cDNA was completed using 150 bp paired-end reads on a NovaSeq X Plus platform. All samples had between 22 to 24.5 million read pairs, and 94% of base pairs had a Q score greater than 30.

### 3.9. Bioinformatics Analysis

Basic sequencing analysis was performed by SeqCenter. Demultiplexing, quality control, and adapter trimming were performed with bcl-convert v4.2.4 (Illumina), and reads were then mapped to the previously sequenced *C. jejuni* S27 genome (NCBI Accession CP131444.1) [34] with HISAT2 v2.2.0 [36] using the ‘—very-sensitive’ parameter. The alignment rate was 68–73%. Reads were quantified using the featureCounts function of Subread v2.0.1 [37] with the ‘-Q 20’ parameter. Read counts were normalized with the Trimmed Mean of M values algorithm of edgeR v1.14.5 [38] in R v4.0.2 [39]. Normalized counts were converted to counts per million, and then differential gene expression analysis was completed using edgeR’s glmQLFTest.

## 4. Conclusions

*Campylobacter* cells exposed to gaseous ClO_2_ were killed rapidly, and transcriptomic results showed that this was likely due to oxidation of critical cellular components. In order to survive under gaseous ClO_2_, cells defend themselves by upregulating canonical oxidative stress response genes *sodB*, *ahpC*, *katA, msrP,* and *trxB*, iron transport systems *ceuBCD*, *cfbpABC*, and *chuBCD*, a phosphate transport system *pstSCAB*, and DNA repair and protection genes *rdgB* and *mutY*. Iron transport genes have previously been noted in oxidative stress responses in *C. jejuni* and other bacteria and may facilitate the oxidative stress response directly by increasing the amount of cellular enterochelin (which may scavenge free radicals) or indirectly by providing sufficient cellular iron to serve as co-factors in other oxidative stress response proteins.

## Figures and Tables

**Table 1:** Primers used in qRT-PCR analysis.

Protein	Function	Gene	Primer	Primer Sequence (5′ -> 3′) [11]
Gyrase subunit A	Housekeeping	*gyrA*	Forward	TGCTAAAGTGCGTGAAATCG
			Reverse	GCATTGGTGCGTTTTCCTAT
Catalase	Oxidative stress response	*katA*	Forward	ACCGTTCATGCTAAGGGAAG
			Reverse	CCTACCAAGTCCCAGTTTCC
Co-chaperonin	General stress response	*groES*	Forward	AAACAACAGCCTCAGGCATAA
			Reverse	TTCTGTTCCACCGTATTTAGCA
Fe^3+^ ABC transporter substrate-binding protein	Iron-uptake ABC transport system	*cfbpA*	Forward	CCACTAATGTTAATATGCGTTCC
			Reverse	TGTGCTTGATAATCTTGCGACAA
RelA/spoT family	General stress response	*spoT*	Forward	GCCCCAATAGCCCATAGAC
			Reverse	ACCCCAAGCAAATCAAGAAC
Chaperone	General stress response	*dnaK*	Forward	CGGTATGCCACAAATCGAAG
			Reverse	GCTAAGTCCGCTTGAACCTG
Alkyl hydroperoxide reductase C	Oxidative stress response	*ahpC*	Forward	AGTTGCCCTTCGTGGTTCGT
			Reverse	ATCGCCCTTATTCCATCCTG
Superoxide dismutase	Oxidative stress response	*sodB*	Forward	TGGCGGTTCATGTCAAAGTA
			Reverse	ACCAAAACCATCCTGAACCA

## Data Availability

Data is contained within the article and Supplementary Material.

## Supplementary Materials

The following supporting information can be downloaded at: https://www.mdpi.com/article/10.3390/ijms26073254/s1.
